# Photocatalytic activity of ZrO_2_/TiO_2_/Fe_3_O_4_ ternary nanocomposite for the degradation of naproxen: characterization and optimization using response surface methodology

**DOI:** 10.1038/s41598-022-14676-y

**Published:** 2022-06-20

**Authors:** Masoud Habibi Zare, Arjomand Mehrabani-Zeinabad

**Affiliations:** Department of Chemical Engineering, Isfahan University of Technology, Isfahan, 84156-83111 Iran

**Keywords:** Environmental sciences, Nanoscience and technology

## Abstract

In this study, ZrO_2_, TiO_2_, and Fe_3_O_4_ components were synthesized by co-precipitation, sol–gel, and co-precipitation methods, respectively. In addition, solid-state dispersion method was used for synthesizing of ZrO_2_/TiO_2_/Fe_3_O_4_ ternary nanocomposite. The ZrO_2_/TiO_2_/Fe_3_O_4_ nanocomposite was characterized by different techniques including XRD, EDX, SEM, BET, FTIR, XPS, EELS, and Photoluminescence (PL). The FTIR analysis of ZrO_2_/TiO_2_/Fe_3_O_4_ photocatalyst showed strong peaks in the range of 450 to 700 cm^−1^, which represent stretching vibrations of Zr–O, Ti–O, and Fe–O. The results of FTIR and XRD, XPS analyses and PL spectra confirmed that the solid-state dispersion method produced ZrO_2_/TiO_2_/Fe_3_O_4_ nanocomposites. The EELS analysis confirmed the pure samples of Fe_3_O_4_, TiO_2_ and ZrO_2_. The EDAX analysis showed that the Zr:Ti:Fe atomic ratio was 0.42:2.08:1.00. The specific surface area, pores volume and average pores size of the photocatalyst were obtained 280 m^2^/g, 0.92 cm^3^/g, and 42 nm respectively. Furthermore, the performance of ZrO_2_/TiO_2_/Fe_3_O_4_ nanocomposite was evaluated for naproxen removal using the response surface method (RSM). The four parameters such as NPX concentration, time, pH and catalyst concentration was investigated. The point of zero charge of the photocatalyst was 6. The maximum and minimum degradation of naproxen using photocatalyst were 100% (under conditions: NPX concentration = 10 mg/L, time = 90 min, pH = 3 and catalyst concentration = 0.5 g/L) and 66.10% respectively. The stability experiment revealed that the ternary nanocatalyst demonstrates a relatively higher photocatalytic activity after 7 recycles.

## Introduction

Environmental pollution by pharmaceutical compounds is considered as one of the most serious issues in recent years^1,2^. For the treatment of pharmaceutical wastewater and for removing pollutants before those are released into the environment, identification of the most efficient method is a challenge^3^. Pharmaceutical wastewater can be treated using physical^4^, chemical, biological^5^, as well as combined methods^6^. The Advanced oxidation processes^7^ including advanced oxidation based on the sulfate radicals^8^, ultraviolet–visible^9^, natural sunlight^10^, Fenton oxidation^11^, electrochemical^12^, nanocomposite catalysts^13^, and sonolysis and sono-Fenton^14^ have been widely used to remove pollutants from pharmaceutical wastewater with great performance. The formation of radicals during these processes leads to the oxidation of organic pollutants in aqueous solutions. In comparison to other methods, photocatalysis offers several advantages, such as high efficiency, low cost, design of suitable catalysts for specific wastewaters, and high corrosion and temperature stability^15–17^. Titanium dioxide (TiO_2_) has been widely used as a catalyst in the degradation of organic compounds and pharmaceutical pollutants since it is a light-sensitive semiconductor (including UV and visible light)^18,19^. The formation of valence band holes and conduction band electrons during photocatalysis produces oxidation–reduction media in wastewater. It can easily degrade organic compounds and convert them into non-toxic compounds such as CO_2_ and water^20,21^. Titanium dioxide is a polymorphic material with three crystalline phases: anatase, rutile, and brookite. The anatase phase is more photocatalytically active than the rutile phase^22,23^. In order to enhance the TiO_2_ photocatalyst activity, it is important to use smaller particles (nano size), as smaller particles have higher specific surface areas^24^. The removal of titanium dioxide nanoparticles after treatment reduces the benefit of this photocatalyst, and immobilization of titanium dioxide (TiO_2_) onto supporting materials can be performed, but immobilization reduces the specific surface area in comparison with a homogenous catalysts^25^. On the other hand, fast recombination of generated electron–hole pairs can decrease the activity of titanium dioxide photocatalyst^26^. Therefore, some other semiconductors such as ZrO_2_ are used to improve of the activity of TiO_2_. ZrO_2_ doping can slow down the electron–hole pair recombination, strengthen the material and increase surface area and anatase to rutile crystal phases ratio^27,28^. ZrO_2_-TiO_2_ photocatalyst has been used to degrade organic compounds, which in this compound, ZrO_2_ acts as support or photocatalyst in the system^29^.

The addition of a small amount of ZrO_2_ to TiO_2_ can increase the surface area because ZrO_2_ inhibits anatase to rutile phase transitions, densification, and crystallite growth by providing dissimilar boundaries^30^. It was observed that changes in surface chemistry, particularly acidity improve the photocatalyst activity^31^. In this binary catalyst, holes trap such as the hydroxyl groups, prevent electron–hole recombination and oxidation–reduction reactions, and increase quantum yield^31^. In addition to ZrO_2_ nanoparticles, it is necessary to be added another compound to these binary metal oxides to improve the surface area of the TiO_2_ photocatalyst as well as recovery of TiO_2_ from treated wastewater^32,33^. Magnetic nanoparticles, such as Fe_3_O_4_, are suitable for this purpose. A Fe_3_O_4_@TiO_2_ photocatalyst has been synthesized to degrade Bisphenol A under visible and long-wavelength UV light irradiation. Moderate iron loading reduced hole-pair separation effectively, and shifted the bandgap in the visible range. Further, the magnetic properties of Fe_3_O_4_ play a critical role in recovering the used catalyst from the solution, which facilitates practical applications of the Fe_3_O_4_@TiO_2_ photocatalyst^34^. Fe_3_O_4_@SiO_2_@g-C_3_N_4_/TiO_2_ nanocomposite was used to remove dye pollutants. It has been found that the 10 wt.% g-C_3_N_4_/TiO_2_ composite catalyst can degrade over 91% of anionic and cationic dyes^35^. Therefore, it is possible to combine the three compounds to degrade toxic and harmful pollutants in wastewater. In recent years, non-steroidal anti-inflammatory drugs (NSAIDs) have been increasingly used for the treatment of COVID-19^36,37^. Therefore, it is predicted that pharmaceutical-produced wastewater containing NSAIDs such as naproxen will increase and it will be very important to develop an effective method of degradation these compounds from the wastewater. The degradation of naproxen by H_2_O_2_-modified titanate nanomaterial and Bi-modified titanate nanobulks under visible light irradiation has been successfully accomplished^38,39^. In previous works, each of these metal oxides was used separately as a catalyst to remove various contaminants^40–43^, but the interaction of the three metal oxides of Fe_3_O_4_, TiO_2_ and ZrO_2_ together and its function to remove pharmaceutical contaminants can produce a synergistic effect. The difference in physical and chemical properties of each component of these nanophotocatalyst makes it unique. A nanocomposite with magnetic, stable, and high photocatalytic properties was finally synthesized^44,45^. TiO_2_ has a band gap of 3.2 eV for anatase, and 3.0 eV for rutile^46,47^. The ZrO_2_ is used as a photocatalyst in heterogeneous reactions due to its semiconductor properties (type n). The band gap energy of ZrO_2_ varies from 3.25 to 5.1 eV depending on the sample preparation^48–55^. Its common band gap energy is 5 eV^55^. It has a wide bandgap and a highly negative flat-band potential, which can be used for hydrogen production through water decomposition^55,56^. Magnetic properties of Fe_3_O_4_ nanoparticles make recyclable nanocomposites possible. Furthermore, the excitation range of nanocomposites synthesized from Fe_3_O_4_ can be used in the visible light range due to their unique optical properties.

The Response surface methodology (RSM) is a widely used mathematical and statistical method for modeling and analyzing a process in which a response is affected by multiple variables, and its goal is to optimize the response^57,58^. Synthesis of Fe_3_O_4_/TiO_2_/ZrO_2_ nanocomposite can improve the photocatalytic property of catalyst. The synthesis of these nanoparticles individually can have some disadvantages such as a wide energy gap, activity only in UV, lower photocatalytic activity, However, their composite synthesis will offer advantages such as higher magnetic properties, recyclable, higher stability and activity, and enhanced photocatalytic activity^59–67^. The Box–Behnken design (BBD) was used in this study due to its rotatable or nearly rotatable second-order design. The percentage of naproxen degradation was selected as the experimental design response^68,69^.

The current work is focused on the synthesis of a new ZrO_2_/TiO_2_/Fe_3_O_4_ nano-photocatalyst with high photocatalytic activity and reusability for the pharmaceutical wastewater treatment. Its performance has been evaluated in the removal of naproxen from pharmaceutical wastewater under UV light. The synthesised photocatalyst has been characterized with methods such as X-ray diffraction (XRD), Energy Dispersive X-Ray (EDX), Scanning Electron Microscope (SEM), Brunauer–Emmett–Teller (BET), Fourier Transform Infrared Spectroscopy (FTIR), X-ray Photoelectron Spectroscopy (XPS), Electron energy-loss spectroscopy (EELS), and Photoluminescence (PL). We investigated various factors including the initial concentration of naproxen, initial pH of the solution, photocatalyst dosage, and time until the optimal degradation efficiency was obtained using the RSM method.

## Experimental and methods

### Materials

Titanium (IV) iso-propoxide 97%, zirconyl(IV) chloride octahydrate (ZrOCl_2_.8H_2_O, 98%), and Iron (II) chloride tetrahydrate (≥ 99%) and iron (III) chloride hexahydrate(≥ 99%) were used as a precursors for the synthesis of TiO_2_, ZrO_2_, and Fe_3_O_4_ respectively. Ethanol was obtained from Sigma-Aldrich and utilized without any further processing. Deionized water (DI-water) was also used in the experiment. Sulphuric acid (H_2_SO_4_, 0.1 M) and sodium hydroxide (NaOH, 0.1 M) were used for the adjustment of the pH of the solution. (S)-6 methoxy α-methyl 2-naphthaleneaceticb sodium salt was purchased from Sigma Aldrich and its aqueous solution used for degradation experiments. In Table 1, naproxen's physical and chemical properties are shown.

**Table 1:** Physical and chemical properties of naproxen.

Property	Value
Molecular structure	
Solubility	15.9 mg/L at 25 °C
Melting point	152–154 °C
Log K_ow_	3.18
pK_a_	4.15

### Synthesis of nanoparticles

#### ZrO_2_ nanoparticles

The co-precipitation method was used for the synthesis of ZrO_2_ nanoparticles. Firstly, ZrOCI_2_.8H_2_O was dissolved in deionized water. Then, 2 M NaOH solution was added to the solution in order to achieve a solution with a pH of 10. The solution was gently stirred for 1 h. After that, the precipitate was filtered, washed with distilled water to reach neutral pH. The obtained powder was dried in an oven at 60 °C for 24 h and then calcined at 700 °C for 10 h^70^.

#### TiO_2_ nanoparticles

Sol–Gel method was used for the preparation of TiO_2_ nanoparticles. 15 mL Titanium (IV) isopropoxide was added into 60 mL ethanol. A magnetic stirrer was used to mix the solution for 30 min. 10 mL deionized water in a drop-wise fashion was added into the mixture until hydrolysis reaction occurred in the system. The obtained white gel was dried at 100 °C and later calcined at 450 °C for 2 h^71^.

#### Fe_3_O_4_ nanoparticles

Known amounts of FeCl_3_.6H_2_O (0.605 g) and FeCl_2_.4H_2_O (0.215 g) were dissolved in deionized water. Then, the obtained solution was placed in an ultrasonic homogenizer for 5 min. After that, 60 mL of 1 M NaOH was added dropwise into solution under ultrasonic at 70 °C. After 60 min, the brownish powder was obtained at a pH of 13.4 and it was separated from the solution using a centrifuge. Finally, it was calcined at 300 °C for 1 h^72^.

#### ZrO_2_/TiO_2_/Fe_3_O_4_ nano-photocatalyst

ZrO_2_/TiO_2_/Fe_3_O_4_ photocatalysts were prepared using solid-state dispersion method. ZrO_2_, TiO_2_, and Fe_3_O_4_ nanoparticles were mixed at a ratio of 4:8:1 in ethanol solution. The resultant ternary oxide was stirred at a constant rate of 300 rpm for 15 min. Then, ethanol was removed from a mixture of nanocomposite by evaporation. Then, the product was dried at 110 °C and calcined at 450 °C for 6 h to obtain ZrO_2_/TiO_2_/Fe_3_O_4_ nanocomposite photocatalyst^60^. The mixing ratio of used components in the synthesized nanocomposite was selected based on the highest efficiency of naproxen degradation under the same operation conditions. The best ratio was achieved (4:8:1) of the ZrO_2_, TiO_2_, Fe_3_O_4_ compounds.

### Photocatalyst characterization

The mineralogical analysis of the prepared nanoparticles was characterized by X-ray diffraction (Inel France, Equinox 3000) with Cu *Kα* radiation. SEM(AIS2100). It was used to determine the surface morphology and microstructure of ZrO_2_/TiO_2_/Fe_3_O_4_ nanocomposite photocatalyst. The Fourier-transform infrared spectroscopy (VERTEX 70, Bruker, USA) in the wavelength range from 400 to 4000 cm^−1^ was used to analyze the chemical bonding in a photocatalyst. Texture properties of nanoparticles was investigated using TriStar-II-Series, Micromeritics Instrument Corporation, USA. The specific surface area of photocatalysts was determined via the N_2_ adsorption–desorption and Brunauer–Emmett–Teller (BET) model. Barrett-Joyner–Halenda (BJH) method was used to calculate the size of pores and pore size distribution using adsorption–desorption curves. Powder samples for XPS analysis are prepared in the form of pellets in the laboratory (the powder should be large enough to cover a surface of 1.5 × 1.5 cm), then the samples should be placed in a vacuum chamber after preparation. A PHI Perkin-Elmer Model 5400 instrument was used to record XPS spectra. The EELS analysis was obtained using a sub-nanometer probe size using the GIF2000 Filter. The PL Spectroscopy was recorded by Avaspec-2048-TEC model spectrometer.

### Point of zero charge determination

An amphoteric oxide method was used to determine the point of zero charge of the photocatalyst^73^. In order to determine the point of zero charge (PZC), 0.25 g of the synthesized photocatalyst was added into the solution with the same ionic strength and different pH values. The synthesized nanocatalyst was added to 50 mL of 0.1 M NaNO_3_ solution. The pH of solution was adjusted in the range of 3–9 using 0.1 M H_2_SO_4_ and 0.1 M NaOH. The pH of solution was measured before and after 24 h mixing and those were called pH_i_ and pH_f_ respectively. The plot of pH changes (ΔpH) as a function of initial pH_i_ was used for the determination of PZC^73^.

### Optimization of the photocatalyst Activity

In order to optimize photocatalyst activity, the RSM has been used. Design Expert 8.0 software (Stat-Ease, Inc., USA) was used to design experiments and analyze mathematical modeling. Each experiment was performed in duplicate. Box–Behnken design (BBD) was applied in this study because of its rotatable or nearly rotatable second-order design. The “y” was the response (the percentage of naproxen degradation). We evaluated four independent variables, including catalyst loading dosage, initial concentration of naproxen, time and pH value in order to find an optimal condition:

1 $$y=f({x}_{1},{x}_{2},{x}_{3},{x}_{4})$$

### Photocatalyst activity

ZrO_2_/TiO_2_/Fe_3_O_4_ Photocatalyst was used for the degradation of naproxen in a pharmaceutical synthetic solution. The experiments were conducted based on the conditions in Table 2. In this study, the effects of solution pH, time, initial naproxen concentration, and photocatalyst loading dose on naproxen degradation were evaluated. To determine the adsorption behaviour of the photocatalyst, the solution kept was in the dark for half an hour. The reactor was irradiated immediately after adding the photocatalyst. For each experiment, a 150 W UV light source has been used for irradiation (The intensity was kept as 15 W/m^2^). At the end of each experiment, centrifugation with 12,000 rpm for 20 min have been performed for each withdrawn sample followed by absorbance measurement using UV‐Vis (Shimadzu UV2401PC) at λ = 230 nm. The efficiency of designed photocatalysts for the degradation of naproxen was calculated as follows:

**Table 2:** Operating parameters for degradation of naproxen experiment.

Factor	Name	Units	Minimum	Maximum	Coded low	Coded high
A	NPX concentration	mg/L	10.00	30.00	− 1 ↔ 10.00	+ 1 ↔ 30.00
B	Time	Min	30.00	90.00	− 1 ↔ 30.00	+ 1 ↔ 90.00
C	pH	–	3.00	9.00	− 1 ↔ 3.00	+ 1 ↔ 9.00
D	Catalyst concentration	g/L	0.1000	0.5000	− 1 ↔ 0.10	+ 1 ↔ 0.50

2 $$NPXR\left(\%\right)=\left(\frac{{C}_{i}-{C}_{f}}{{C}_{i}}\right)\times 100$$

where C_i_ and C_f_ are the naproxen concentration before and after degradation reaction respectively.

### Reusable photocatalytic properties

Reusability tests were conducted for ZrO_2_/TiO_2_/Fe_3_O_4_ Photocatalyst. Briefly, the selected photocatalyst being used in the degradation test of naproxen was separated from solution using a 1.3 Tesla magnet. Used photocatalysts were rinsed in distilled water and irradiated under UV light for 12 h. In order to evaluate the reusability of a photocatalyst, seven successive experimental runs were conducted.

## Results and discussion

### Characterization

#### XRD analysis

**Figure 1:**
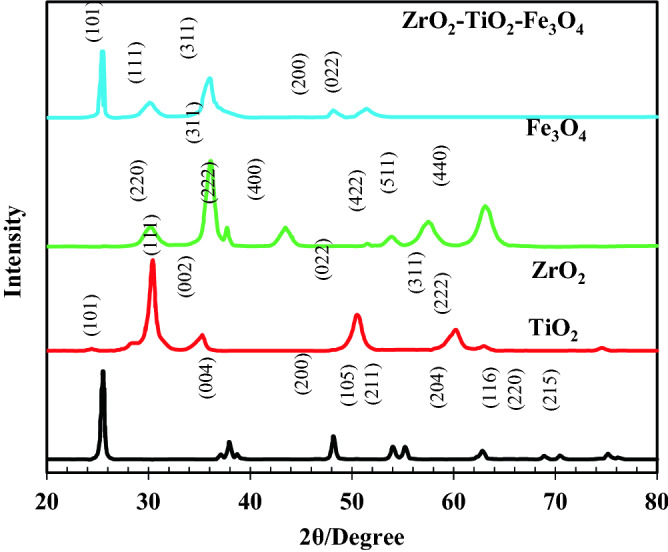
The XRD patterns of ZrO_2_, TiO_2_, Fe_3_O_4_, and ZrO_2_/TiO_2_/Fe_3_O_4_ nanocomposite.

The constituting phase, crystalline size, and crystalline structures of the ZrO_2_, TiO_2_, and Fe_3_O_4_, nanoparticles, and ZrO_2_/TiO_2_/Fe_3_O_4_ nanocomposite characterized using XRD, which are depicted in Fig. 1. For ZrO_2_, the primary characteristic diffraction peaks appeared at 2θ = 30°, 35°, 50.8°, 60.1°, and 63° were corresponds to crystal planes of (1 1 1), (0 0 2), (0 2 2), (3 1 1), and (2 2 2). The majority of these crystalline phases are monoclinic. The transition from tetragonal to monoclinic can occur with an increase in the calcination temperature which increases the crystallite size of the samples^74^. According to Fig. 1 for TiO_2_ sample, there is good agreement between the results of this study and the XRD pattern for the titanium dioxide phase reported in the literature^75–77^. The diffraction peaks at 2 thetha values of 25.32°, 37.90°, 48.09°, 54.10°, 55.15°, 62.85°, 68.99°, 70.49°, and 75.12° were belonged to crystal planes of (1 0 1), (0 0 4), (2 0 0), (1 0 5), (2 1 1), (2 0 4), (1 1 6), (2 2 0), (2 1 5)^78^. All the peaks observed in the diffraction pattern of TiO_2_ nanoparticles are in good agreement with anatase (JCPDS No. 00–001-0562). Figure 1 clearly shows magnetite formation with well-defined crystallinity in Fe_3_O_4_. All of the diffraction peaks are related to (2 2 0), (3 1 1), (2 2 2), (4 0 0), (4 2 2), (5 1 1), and (4 4 0) crystal planes and those were belonged to Fe_3_O_4_. The results confirmed the occurrence of inverse spinel structure in Fe_3_O_4_. The XRD peaks of impurities were not observed, which means that the synthesized Fe_3_O_4_ nanoparticles are pure^79^. Finally, in the XRD pattern of ZrO_2_/TiO_2_/Fe_3_O_4_ nanocomposite, the main peaks related to all three compounds were observed. The results showed that the sample has spinal reverse cube of Fe_3_O_4_ structure. The ZrO_2_ is amorphous phase and the phase of TiO_2_ is tetragonal (Anatase). The average crystallite sizes were calculated by Scherrer formula:

3 $${\text{D}} = \frac{{0.9 \times {\uplambda }}}{{{\upbeta } \times \cos {\uptheta }}}$$

where λ is X-ray wavelength, β is the full width at half maximum of the diffraction line and θ is the diffraction angle^80^. The average crystallite size of TiO_2_, ZrO_2_, Fe_3_O_4_ and ZrO_2_/TiO_2_/Fe_3_O_4_ was found to be 18.75 nm, 22.29 nm, 13.27 and 33.42 nm respectively. The most important cause of difference in particle size of synthesized samples can be the difference in their synthesis method^81^.

#### EDX analysis

**Figure 2:**
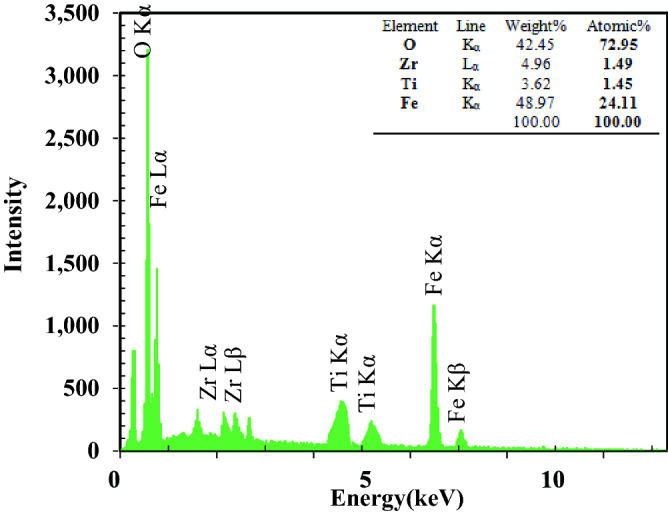
EDX spectrum. (Insert: Element analysis of synthesized nanocomposite).

EDAX analysis confirmed the presence of all elements, including Zr, Ti, O and Fe in the synthesized photocatalyst and there is no evidence of any other element. The inserted table in Fig. 2 presents an elemental composition of the photocatalyst. The results showed that the atomic ratio of Zr:Ti:Fe was 0.42:2.08:1.00. The synthesized nanocomposite had different peaks at 0.70, 6.39, and 6.92 keV which are related to L_α_, K_α_, and K_β_ of Fe and 0.52 keV is K_α_ of oxygen. Peaks at 2.04 (L_α_) and 2.26 (L_β_) keV are for zirconium. Fe_3_O_4_ particles are larger than TiO_2_ particles based on the results obtained.

#### SEM analysis

**Figure 3:**
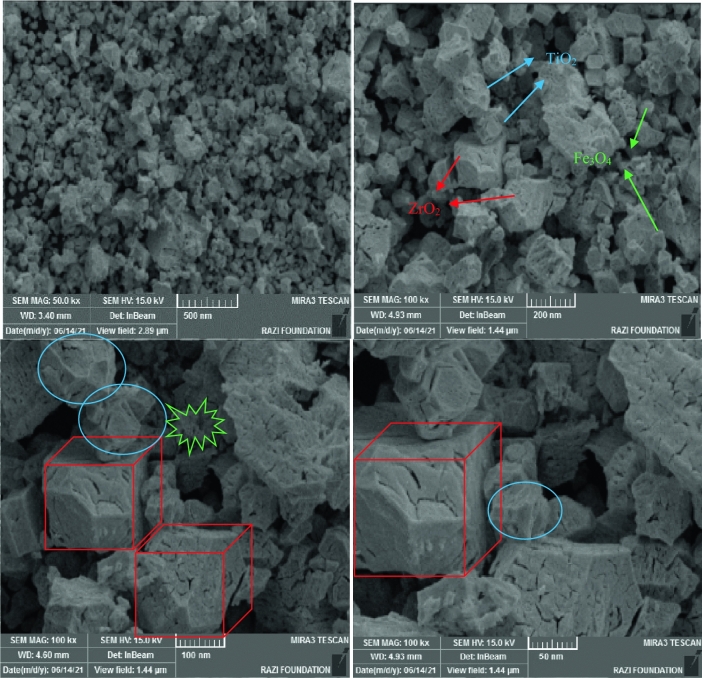
SEM image of ZrO_2_/TiO_2_/Fe_3_O_4_ nanocomposite.

SEM analysis was used to determine the morphology and size of nanoparticles of ZrO_2_/TiO_2_/Fe_3_O_4_ nanocomposite (see in Fig. 3). In Fig. 3, ZrO_2_ and TiO_2_ nanoparticles are almost cubic and spherical respectively, while Fe_3_O_4_ does not have a specific shape.

#### BET analysis

**Figure 4:**
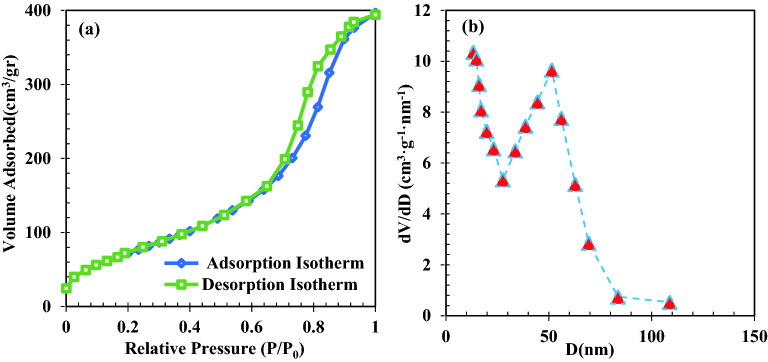
(**a**) N_2_ adsorption–desorption hysteresis and (**b**) pore size distribution of ZrO_2_/TiO_2_/Fe_3_O_4_ photocatalyst.

Figure 4a and b show the porosity distribution curve and pore size distribution of ZrO_2_/TiO_2_/Fe_3_O_4_ nanocomposite. The specific surface area (SSA) was calculated using the N_2_ isotherms. The BET-SSA, the volume of pores, the average size of pores of the photocatalyst obtained were 280 m^2^/g, 0.92 cm^3^/g, and 42 nm respectively. The sample is mesoporous as the diameter of pores is larger than 2 nm. Hysteresis loop of types IV-H1 was obtained for the sample which is related to cylindrical and spherical pores in the sample^82^. In terms of pore size distribution, wide pore size distribution was observed, especially, pores with size larger than 3 nm had significant volume in the sample. It was found that the pore size larger than 3 nm is appropriate for the reactants penetration into the porous media of photocatalyst^83,84^.

#### FTIR analysis

**Figure 5:**
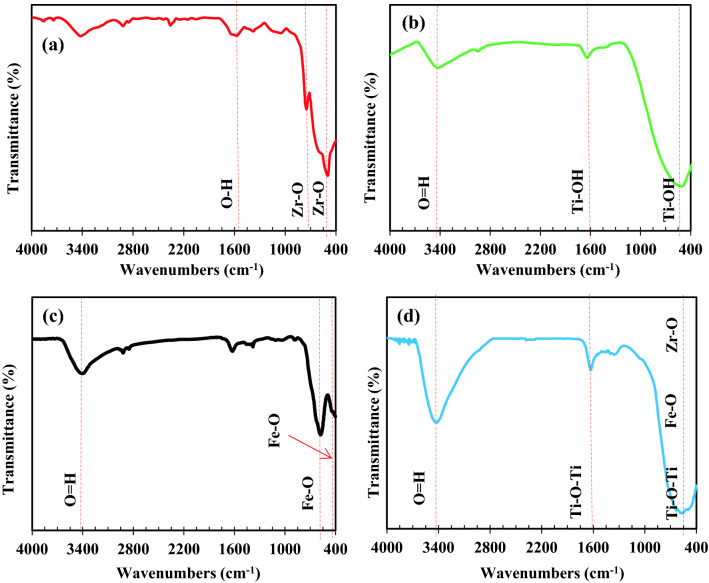
FTIR spectra of (**a**) ZrO_2_, (**b**) TiO_2_, (**c**) Fe_3_O_4_, (**d**) of ZrO_2_/TiO_2_/Fe_3_O_4_ photocatalyst.

Figure 5a–d shows the FTIR analysis of ZrO_2_, TiO_2_, Fe_3_O_4_, and ZrO_2_/TiO_2_/Fe_3_O_4_ photocatalysts. As for ZrO_2_, the strong broad peak at 498–502 cm^−1^ region is attributed to the vibration mode of ${\mathrm{ZrO}}_{3}^{2-}$ groups. The peaks around 754 cm^−1^ are related to the Zr–O stretching vibrations in ZrO_2_. The IR at 1553 cm^−1^ attributed to stretching of O–H groups, which indicates the adsorbed moisture^85^. For TiO_2_ nanoparticles, TiO_2_ network bonds and deformation vibrations of stretching mode of Ti–OH peaks were observed at 483 cm^−1^ and 1623 cm^−1^ respectively. It can be related to the absorption of water on the TiO_2_ surface. Asymmetrical and symmetrical stretching vibration of hydroxyl groups (–OH) was observed at 3405 cm^−1^. The obtained results are consistent with those reported in the literature^86,87^. According to Fig. 5c, the stretching vibration of the O–H can be attributed to the absorption band 3444 cm^−1^. The peaks at 419 cm^−1^ and 589 cm^−1^ were attributed the stretching vibration of the Fe–O. The strong peaks in the 450–700 cm^−1^ range were attributed to stretching vibrations of Zr–O, Ti–O, and Fe–O in ZrO_2_/TiO_2_/Fe_3_O_4_ photocatalyst. The results confirm the presence of all three nanoparticles in the ZrO_2_/TiO_2_/Fe_3_O_4_ photocatalyst.

#### XPS analysis

**Figure 6:**
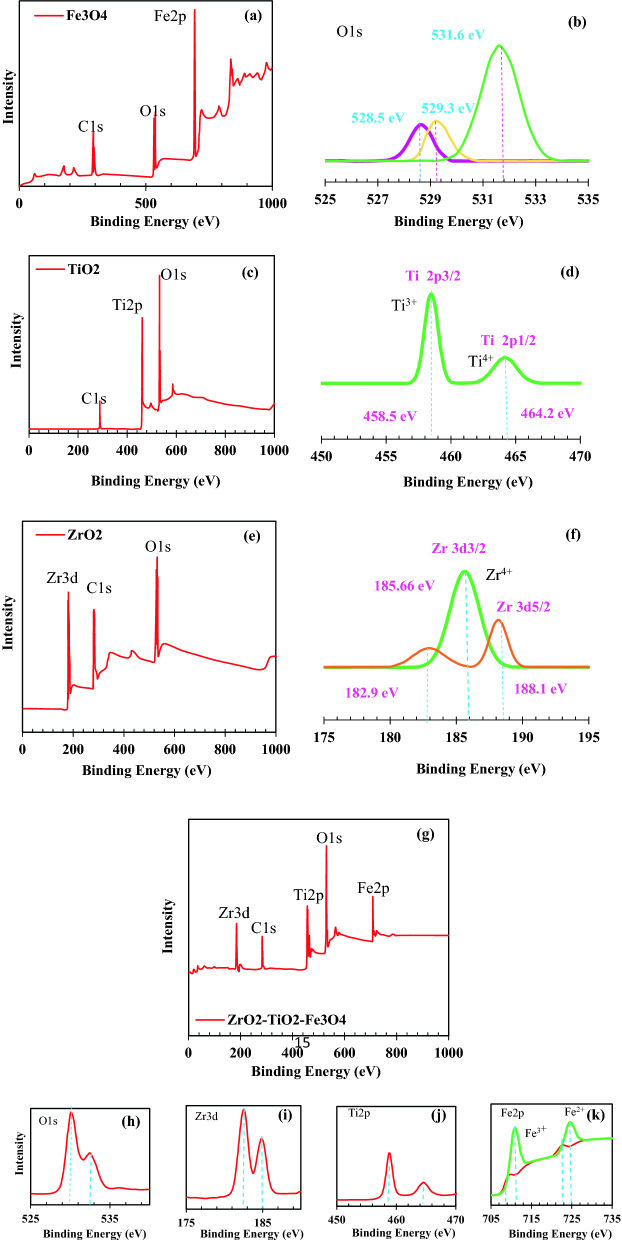
XPS analysis of Nanoparticles (**a**) Fe_3_O_4_, (**b**) The O1s level spectrum, (**c**) TiO_2_, (**d**) The Ti 2p level spectrum, (**e**) ZrO_2_, (**f**) The Zr 3d level spectrum, and (**g**) Nanocomposite of ZrO_2_/TiO_2_/Fe_3_O_4_, XPS spectrum of (**h**) The O1s level (**i**) The Zr 3d level (**j**) The Ti 2p level and (k) The Fe 2p level.

For further investigation of the nature of the synthesized nanomaterial, we performed high-resolution X-ray photoelectron spectroscopy (XPS) on the synthesized ZrO_2_, TiO_2_ and Fe_3_O_4_ pure nanoparticles and the ZrO_2_/TiO_2_/Fe_3_O_4_ nanocomposite. The XPS spectra of pure and nanocomposite samples are shown in Fig. 6. Figure 6a and b show the XPS results of high-resolution Fe_3_O_4_ nanoparticles. Three components of the Fe–O bond can be seen in the XPS spectrum. After photoexcitation, the splitting of O1s at 531.6 eV indicates the formation of a Fe–O bond. The XPS peak of C1s at 292.6 eV and O1s at 531.6 eV indicate the formation of Fe–C and Fe–O bonds following particle photoexcitation (see Fig. 6a and b)^88^. Figure 6c and d show the XPS analysis of the pure TiO_2_ elemental composition. According to the XPS spectrum, elements Ti, C, and O are present on the surface of the TiO_2_ nanoparticles. Fe_3_O_4_ and TiO_2_ nanoparticle samples contained carbon resulting from carbon-based contaminants. The XPS peaks of Fig. 6c show C1s at 288.27 eV and O1s at 532.1 eV and Ti2p at 461.1 eV. Figure 6d shows the peaks of 458.5 eV and 464.2 eV, which correspond to Ti 2p3/2 and Ti 2p1/2, respectively. The peak position between Ti 2p3/2 and Ti 2p1/2 at 464.2 eV indicates the presence of Ti^4+^ oxidation state^89,90^. Figure 6e and f show the XPS spectrum of ZrO_2_ nanoparticles, which indicate the presence of relevant elements. The Zr3d level spectra of Fig. 6f show the Zr3d5/2 and Zr3d3/2 peaks at binding energies of 185.66 eV and 188.1 eV, respectively. The energy difference of 2.44 eV between the two peaks indicates the presence of Zr^+4^^91^. The third suitable peak for the shoulder that appears at the base of Zr 3d3/2 can be attributed to the lack of oxygen, which can be due to under-coordinated Zr sites of very small ZrO_2_ nanoparticles^92^. XPS analysis was investigated to accurately determine the surface composition and chemical state of ZrO_2_/TiO_2_/Fe_3_O_4_ nanocomposite (As shown in Fig. 6g–k). As shown in Fig. 6g, the major peaks of Zr3d, C1s, Ti2p, O1s, and Fe2p are shown at 184.33, 282.59, 458.05, 530.18, and 708.37 eV, respectively. These results, in addition to confirming the presence of three nanoparticles ZrO_2_, Fe_3_O_4_ and TiO_2_, showed that ZrO_2_, Fe_3_O_4_ and TiO_2_ are mainly present as separate phases in the ZrO_2_/TiO_2_/Fe_3_O_4_ composite. Figure 6h shows that the binding energy of O1s appears at 530.08 eV, which proves the existence of oxygen in the crystal lattice (O_2_)^93^. In Fig. 6i, the dual peaks of Zr 3d with binding energies at 182.52 eV and 184.87 eV correspond to the chemical states Zr 3d3/2 and Zr 3d5/2, respectively, indicating zirconium in the + 4 oxidation state^94^. In addition, two peaks at 182.52 and 184.87 eV indicate the presence of Zr-Ti chemical bonds in the composites^95^. In Fig. 6j, the Ti 2p peaks at 458.84 eV and 464.58 eV correspond to the chemical states Ti 2p1/2 and Ti 2p3/2, respectively. Ti peaks confirm the presence of Ti^4+^ oxidation state in the nanocomposite. Meanwhile, the peak of 464.58 eV can be attributed to Zr-Ti chemical bonds^96^. The presence of chemical bonds Fe–O, Zr-O, Ti–O and Zr-Ti, Ti-Fe and Zr-Fe indicates a phase contact between Fe_3_O_4_, TiO_2_ and ZrO_2_. In Fig. 6k for Fe_3_O_4_, the major peaks at 710.96 eV and 724.59 eV are attributed to Fe^3+^ 2p3/2 and Fe^3+^ 2p1/2, respectively. The bond energy of Fe^2+^ 2p3/2 and Fe^2+^ 2p1/2 has a dual peak at 708.96 eV and 721.52 eV, respectively. Results of Fe_3_O_4_ XPS analysis are consistent with Fe2p spectrum^97^.

#### EELS analysis

**Figure 7:**
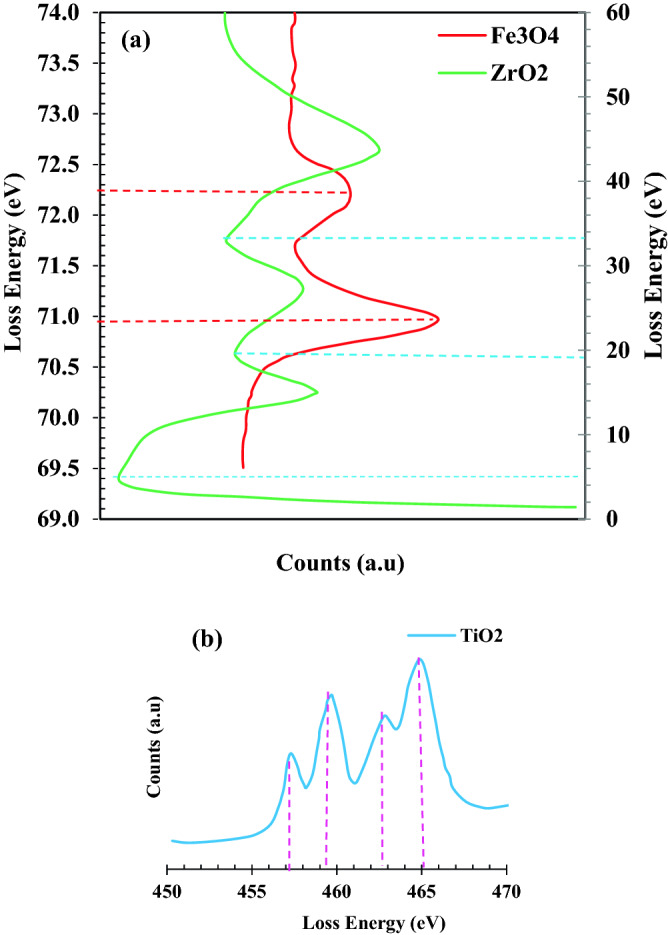
EELS spectrum for Structures of (**a**) Fe_3_O_4_, (**b**) ZrO_2_ and TiO_2_.

The electron energy-loss spectroscopy (EELS) analysis was used to identify the pure samples more accurately. The combination of EELS and statistical analysis can provide more information on the differentiation of Fe_3_O_4_ and Fe_2_O_3_ spinel structures. Figure 7a shows that the energy-loss peaks at 70.96 eV and 72.19 eV can only be attributed to Fe_3_O_4_ phase, since the expected value for gamma- Fe_2_O_3_ (70.2 eV) is significantly different. As shown in Fig. 7a, the EELS spectra of ZrO_2_ nanoparticles are obtained (obtained peaks for ZrO_2_ are 1.4, 14.8, 27.28 and 43.72 eV respectively). The synthesized ZrO_2_ nanoparticles can be proved according to the obtained peaks from the analysis. As shown in Fig. 7, the Ti structure in the EELS spectrum for Ti^4+^, indicates a lower oxidation state which has edges shift slightly towards the lower energy loss. Here, the blue curve is the fine structure of the Ti^4+^ state. EELS was obtained using a sub-nanometer probe size using the GIF2000 Filter.

#### Photoluminescence spectra

**Figure 8:**
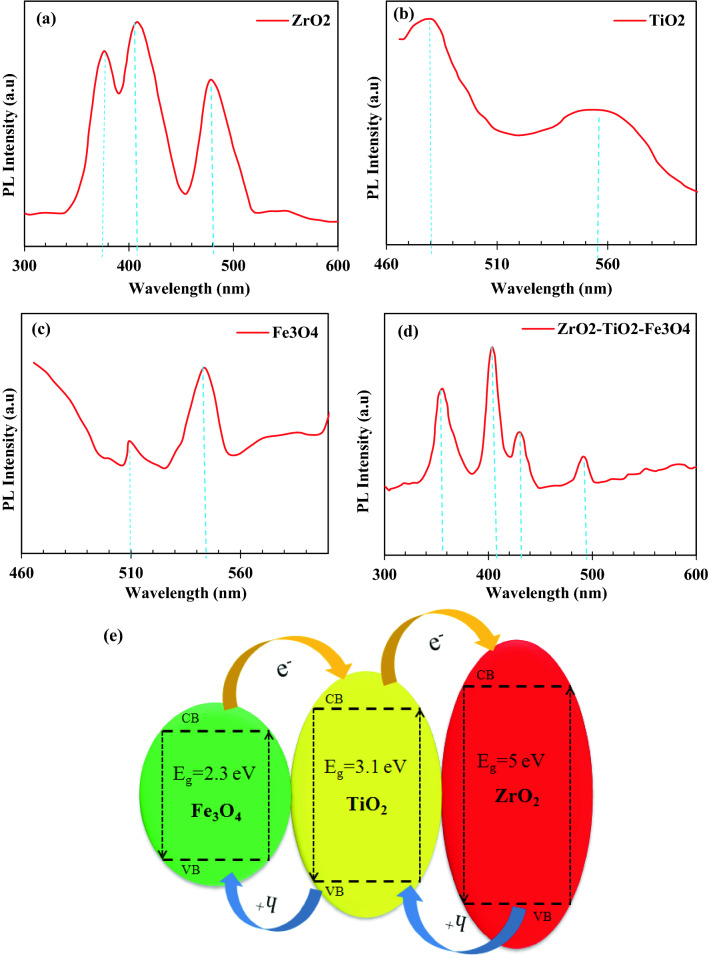
Photoluminescence (PL) spectra of (**a**) ZrO_2,_ (**b**) TiO_2,_ (**c**) Fe_3_O_4_ nanoparticles and (**d**) ZrO_2_/TiO_2_/Fe_3_O_4_ nanocomposite, (**e**) Energy level and electron–hole pair separation/transfer in ZrO_2_/TiO_2_/Fe_3_O_4_ nanocomposite.

The light absorption of photocatalysts significantly affects the activity of photocatalysts. Also, the spectrum of PL (Photoluminescence) and their intensity are closely related to its photocatalytic activity. Figure 8a–d shows the photoluminescence spectra (PL) of pure ZrO_2_, TiO_2_ and Fe_3_O_4_ samples and the ZrO_2_/TiO_2_/Fe_3_O_4_ nanocomposite at room temperature in the wavelength range of 300–600 nm with an excitation wavelength of 293 nm. As shown in Fig. 8a–c the pure ZrO_2_, TiO_2_ and Fe_3_O_4_ samples show significant PL signals at 376, 407, 478, and 480, 556, 509 and 543 nm, respectively. Among all samples, the TiO_2_ sample shows the highest intensity. The obtained PL spectrum for pure ZrO_2_ nanoparticles is almost similar to the results previously reported^98^. According to previous research^99–102^, the presence of these peaks in the visible range is probably due to the presence of oxygen vacancies, defects, surface states and other structural impurities. A high PL intensity of pure ZrO_2_ appears to indicate that the ZrO_2_ surface states are much lower, hence easy electron transfer can occur between VB and CB of ZrO_2_ even with a low-energy laser excitation source (325 nm)^103^. Additionally, when Fe_3_O_4_ and TiO_2_ are loaded with ZrO_2_ as ternary oxide catalysts, the PL intensity of ZrO_2_ decreases abruptly (Fig. 8d). The formation of a chemical interaction between pure oxides when they accumulate together (–Ti–O–Fe–), (–Ti–O–Zr–) and (–Fe–O–Zr–), as shown, has a profound effect on the photocatalytic properties^104^. A PL spectrum of the physically mixed pure ZrO_2_, TiO_2_, and Fe_3_O_4_ catalysts in different ratios further confirms the synergistic effect of the ZrO_2_/TiO_2_/Fe_3_O_4_ ternary system and the presence of (–Ti–O–Fe–), (–Ti–O–Zr–) and (–Fe–O–Zr–). The peaks shown at 403, 427, and 490 nm under the excitation wavelength of 293 nm for the ZrO_2_/TiO_2_/Fe_3_O_4_ nanocomposite must be due to the interference of trap states between the gaps, such as surface defects and oxygen vacancies (Fig. 8d)^105^. Preparation for solid samples (powder) was performed for PL (Photoluminescence Spectroscopy) analysis. The PL Spectroscopy was recorded by Avaspec-2048-TEC model spectrometer. The valence band (VB) and conduction band (CB) are shown in Fig. 8e. In this figure, the photocatalyst energy bands of Fe_3_O_4_/TiO_2_/ZrO_2_ are shown in (CB) and (VB). The VB determines the energy levels of electrons in the VB of an atomic structure. The combination of three metal oxides can be improved electromagnetic separation and narrowing of the photocatalyst band gap. In Fe_3_O_4_/TiO_2_/ZrO_2_ semiconductor, electrons migrate to CB whereas; positive holes are created on VB. The OH is formed through the reaction of charge carriers with adsorbed compounds on the photocatalyst surface. In Fe_3_O_4_/TiO_2_/ZrO_2_ nanocomposite, the electron–hole separation may occur between ZrO_2_ and TiO_2_. The surface energy of Fe_3_O_4_ for VB and CB was placed in TiO_2_ band gap and surface energy of TiO_2_ was placed in ZrO_2_ band gap (Fig. 8e). In the excitation of electrons from three catalysts, the majority of electrons migrate from CB of ZrO_2_ to CB of TiO_2_, then to CB of Fe_3_O_4_. As a result, electron–hole recombination is prevented in ZrO_2_ and occurs in Fe_3_O_4_.

### Photocatalyst performance

The experimental work was designed and the obtained actual results, as well as predicted values, were provided in Table 3. Box–Behnken design for RSM was used for the experimental design. Statistical investigation of experimental data was performed using linear, two-factor interaction, quadratic, and cubic models. The results were given in Table 4. According to Table 4 and R^2^ values, the quadratic model was found the most accurate model for the prediction of experimental data in terms of naproxen removal. Its predicted R^2^ value was found to be 0.9901 which means the developed model equation is able to predict the results accurately. Therefore, the following quadratic polynomial model in terms of coded factors was proposed for investigation of naproxen degradation from pharmaceutical synthetic solution as a function of operating parameters:

4 $$\begin{aligned} & {\mathbf{Sqrt}}\left( {{\mathbf{NaproxenRemoval}}} \right) = - 0.2384{\text{A}} + 0.1918{\text{B}} - 0.1575C \\ & \quad + 0.{359}0{\text{D}} - 0.0{13}0{\text{AB}} - 0.0{\text{836AC }} \\ & \quad + 0.0{\text{471AD}} + 0.0{\text{192BC}} - 0.00{\text{91BD}} \\ & \quad - 0.0{2}0{\text{4CD}} - 0.{\text{1389A}} - 0.{\text{1168B}} - 0.{2}0{\text{35C}} + 0.{161}0{\text{D}} \\ \end{aligned}$$

where A, B, C, D are photocatalyst dose (g/L), time (min), solution initial pH, and initial concentration of naproxen (mg/L). The variables were defined at three levels including − 1, 0, and + 1.

**Table 3:** Operational parameters range in the designed experiments and actual and predicted results in terms of naproxen removal.

Run	Factor 1	Factor 2	Factor 3	Factor 4	Response	
	A:NPX concentration	B:Time	C:pH	D:catalyst concentration	Naproxen Removal	
					Actual Value	Predicted Value
	mg/L	min	–	g/L	%	%
1	10	90	3	0.1	87.3	87.6
2	10	90	9	0.5	96.8	97.2
3	30	90	9	0.1	72.1	72.8
4	10	60	6	0.3	92.3	91.8
5	20	60	3	0.3	88.8	89.1
6	20	60	6	0.3	89.8	89.9
7	20	60	6	0.3	90.5	89.9
8	20	60	6	0.3	89	89.9
9	30	90	3	0.5	95.4	95.3
10	20	60	6	0.1	86.8	86.1
11	30	30	9	0.5	79.2	79.2
12	10	30	9	0.5	88.9	88.9
13	10	30	3	0.5	93.5	93.1
**14**	**10**	**90**	**3**	**0.5**	**100**	**100**
15	20	60	6	0.3	89.6	89.9
16	30	30	9	0.1	66.1	65.8
17	30	30	3	0.1	73.8	73.8
18	10	30	9	0.1	77.5	77.8
19	30	90	3	0.1	79.9	79.7
20	30	30	3	0.5	89.6	89.5
21	30	60	6	0.3	82.8	83.0
22	10	30	3	0.1	80.5	80.5
23	10	90	9	0.1	86.5	86.3
24	20	60	6	0.3	90.2	89.9
25	20	60	6	0.3	89.3	89.9
26	30	90	9	0.5	86.3	86.1
27	20	30	6	0.3	83.8	84.1
28	20	60	9	0.3	83.8	83.2
29	20	60	6	0.5	99.8	100
30	20	90	6	0.3	92.1	91.4

Significant values are in bold.

**Table 4:** Statistical investigation based on linear, two-factor interaction, quadratic, and cubic models.

Source	Sequential *p*-value	Lack of fit *p*-value	Adjusted R^2^	Predicted R^2^	
Linear	< 0.0001	0.0002	0.8158	0.7680	
2FI	0.6041	0.0002	0.8050	0.5781	
Quadratic	** < 0.0001**	**0.5274**	**0.9952**	**0.9901**	**Suggested**
Cubic	0.5874	0.3447	0.9948	0.9248	Aliased

Significant values are in bold.

**Figure 9:**
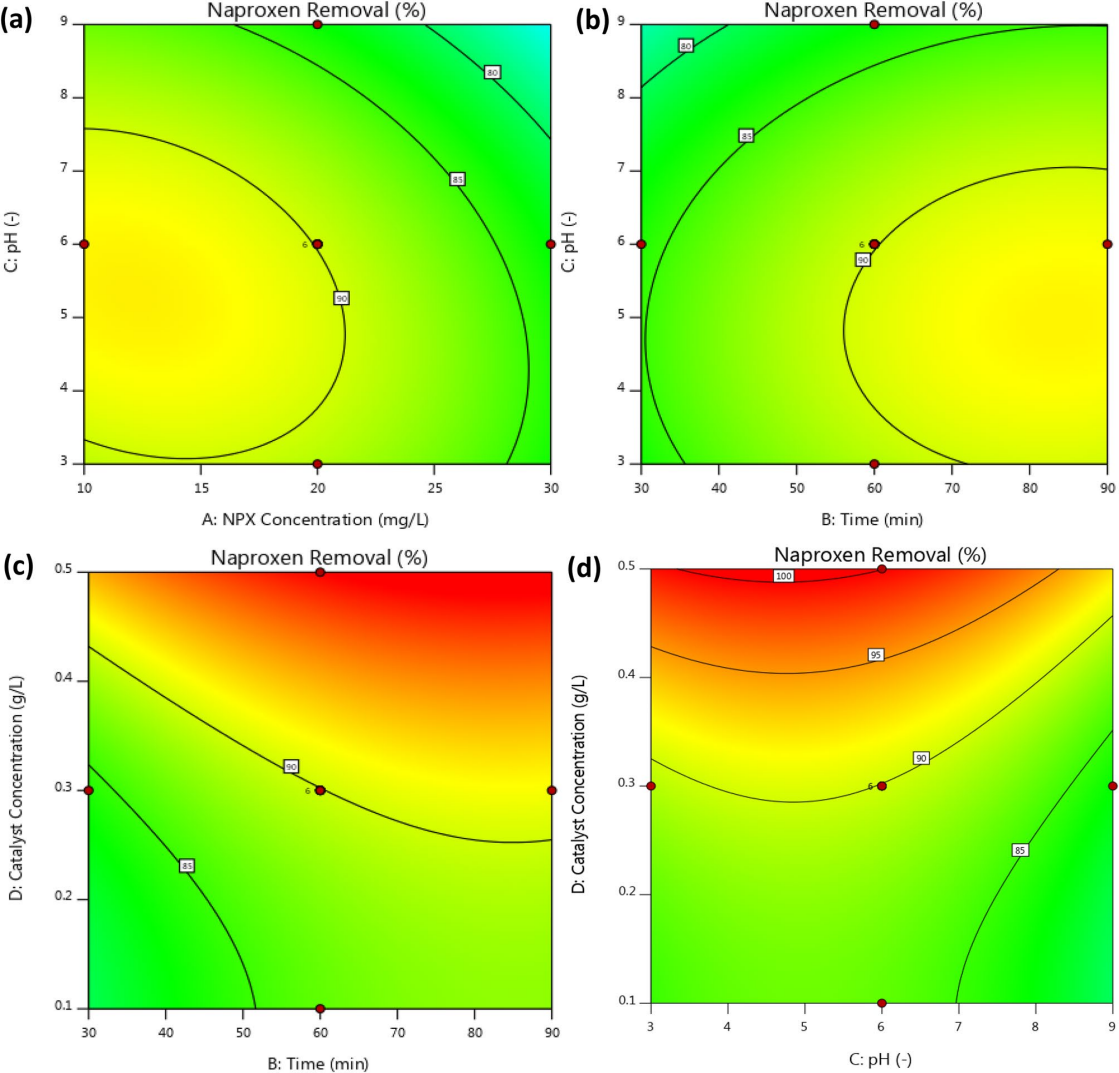
The 2D contour plots of RSM for investigation the degradation percentage of naproxen.

Figure 9 illustrates the individual effects of operational parameters on naproxen removal. The contour lines represent lines of equal response and can be visualized as response contours two factors at a time. In this study, contour lines mean the performance of photocatalyst for naproxen removal at different operating parameters. The contour map reflects the cross-interaction between two variables by keeping the other variable constant. Interaction of initial pH and naproxen concentration for photocatalyst dose of 0.3 g/L and time of 60 min was shown in Fig. 9a. At a pH value of 6, increasing the initial concentration of naproxen from 10 to 30 mg/L led to the reduction of naproxen removal from 91.81 to 82.9%. Naproxen removal is reduced due to solution turbidity and a decrease in light absorption by the photocatalyst. Also, the amount of hydroxyl radical in the solution decreases. Furthermore, the photocatalyst surface is covered by pollutants with increasing its concentration from 10 to 30 mg/L which decreases photon penetration into the photocatalyst. Therefore, the photocatalyst was unable to generate enough electron–hole pairs, resulting in a reduced removal of naproxen. In the interaction between time and initial pH of the solution (Fig. 9b), the photocatalyst dosage and initial concentration of naproxen were considered 0.3 g/L and 20 mg/L respectively. Firstly, the efficiency of naproxen degradation was increased from 90.10 to 91.34% with increasing pH of the solution from 3 to 6, then, it was decreased to 84.9% at a pH value of 9. These changes are related to the oxidation potential and surface charge of the photocatalyst. The pH of the media can have a significant impact on the adsorption and desorption of pollutants on the photocatalyst surface.

**Figure 10:**
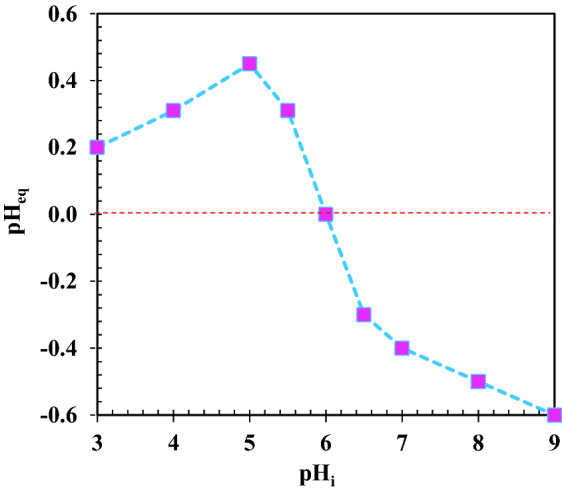
Point of zero charge ZrO_2_/TiO_2_/Fe_3_O_4_ photocatalyst.

The amount of pK_a_ is 4.15 for the naproxen. At pH values higher than 4.15, naproxen has a negative charge, otherwise, it has a positive charge. The neutral charge point of photocatalyst is 6. Therefore, it has a positive charge at pH below 6, whereas the surface charge of photocatalyst is negative at pH above 6. When the pH value is between 4.15 and 6, the naproxen and photocatalyst have opposite charges, so the adsorption of naproxen on the photocatalyst surface increases in this range. It is for this reason that maximum degradation occurs at pH 6 rather than pH 3 or 9. When the pH solution was 6, an increase in time from 30 to 60 min increased naproxen removal from 84.10 to 91.34%. The time and photocatalyst dosage interaction at constant initial pH and concentration of naproxen of 6 and 20 mg/L was shown in Fig. 9c. In Fig. 9c, and for a 60-min experiment run, there was an increase in naproxen degradation from 86.2 to 100% when the photocatalyst dosage was increased from 0.1 to 0.5 g/L. Photocatalyst dosage had a significant effect on naproxen degradation, increasing the number of available active sites for the generation of hydroxyl radicals. However, too much photocatalyst in the system can reduce naproxen degradation as a result of agglomeration of photocatalyst nanoparticles. The reduction in active site of the photocatalyst resulted from agglomeration. It was found that a decrease in pH can increase naproxen degradation (Fig. 9d), and the greatest degradation was obtained at pH 3. The solution pH had a complicated effect on the photocatalytic oxidation reaction. The optimum pH value is highly dependent on the type of pollutant and point of zero charges (PZC) of the photocatalyst. According to Fig. 10, it is 6 for the synthesized photocatalyst. At pH = pH_PZC_, the surface charge is neutral. The surface charge can be positive or negative at pH < pH_PZC_ and pH > pH_PZC_. The amount of adsorption is highly dependent on the surface charge of photocatalyst and pollutant and it can be controlled by a change in solution pH.

**Figure 11:**
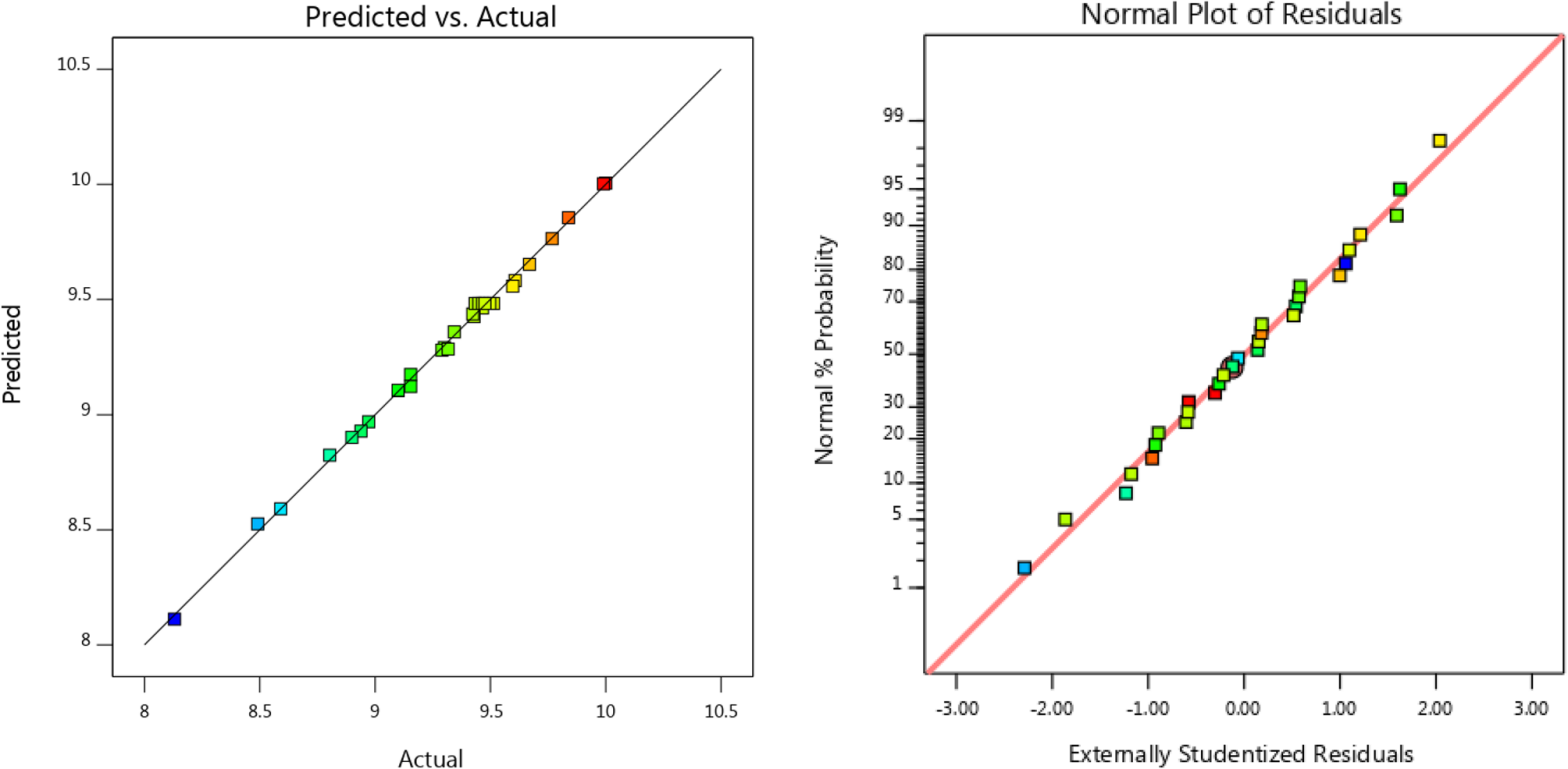
(**a**) The relationship of predicted and actual values of the RSM model for naproxen removal; (**b**) the externally studentized residuals versus normal % probability distribution.

The analysis of variance (ANOVA) results obtained for the present model are summarized in Table 5. The results suggested that a quadratic model is significant because of its high F-value (431.34) and very low *p*-value (< 0.001). The calculated higher F-value and lower *p*-value for the photocatalyst dosage (D factor) in comparison with other factors indicated that the photocatalyst dosage is the most important parameter in the system. The results in Table 5 showed that the proposed statistical model is accurately fitted to experimental data. The accuracy of the proposed model should be investigated in detail. Figure 11 shows a linear relationship between experimental data and predicted values in terms of naproxen removal and it confirms the accuracy of the proposed model as all the data accumulated around a 45-degree line. The normal probability plot of the residuals was shown in Fig. 11b and it can be clearly seen a linear scattering of modeling data. The linear scattering means normal distribution of errors in a defined matrix for experimental design. Therefore, the probability of random error intervention and effect of the sequence of experiments is considerably decreased in the proposed model.

**Table 5:** ANOVA results for the quadratic model.

Source	Sum of Squares	df	Mean Square	F-value	*p*-value	
Model	5.28	14	0.3770	431.34	< 0.0001	Significant
A-NPX Concentration	1.02	1	1.02	1170.41	< 0.0001	
B-Time	0.6622	1	0.6622	757.51	< 0.0001	
C-pH	0.4464	1	0.4464	510.73	< 0.0001	
D-Catalyst Concentration	2.32	1	2.32	2654.05	< 0.0001	
AB	0.0027	1	0.0027	3.09	0.0990	
AC	0.1119	1	0.1119	128.00	< 0.0001	
AD	0.0355	1	0.0355	40.67	< 0.0001	
BC	0.0059	1	0.0059	6.75	0.0202	
BD	0.0013	1	0.0013	1.50	0.2391	
CD	0.0067	1	0.0067	7.61	0.0146	
A^2^	0.0500	1	0.0500	57.22	< 0.0001	
B^2^	0.0353	1	0.0353	40.41	< 0.0001	
C^2^	0.1073	1	0.1073	122.76	< 0.0001	
D^2^	0.0672	1	0.0672	76.84	< 0.0001	
Residual	0.0131	15	0.0009			
Lack of Fit	0.0088	10	0.0009	1.02	0.5274	Not significant
Pure Error	0.0043	5	0.0009			
Cor Total	5.29	29

### Optimization using RSM

**Figure 12:**
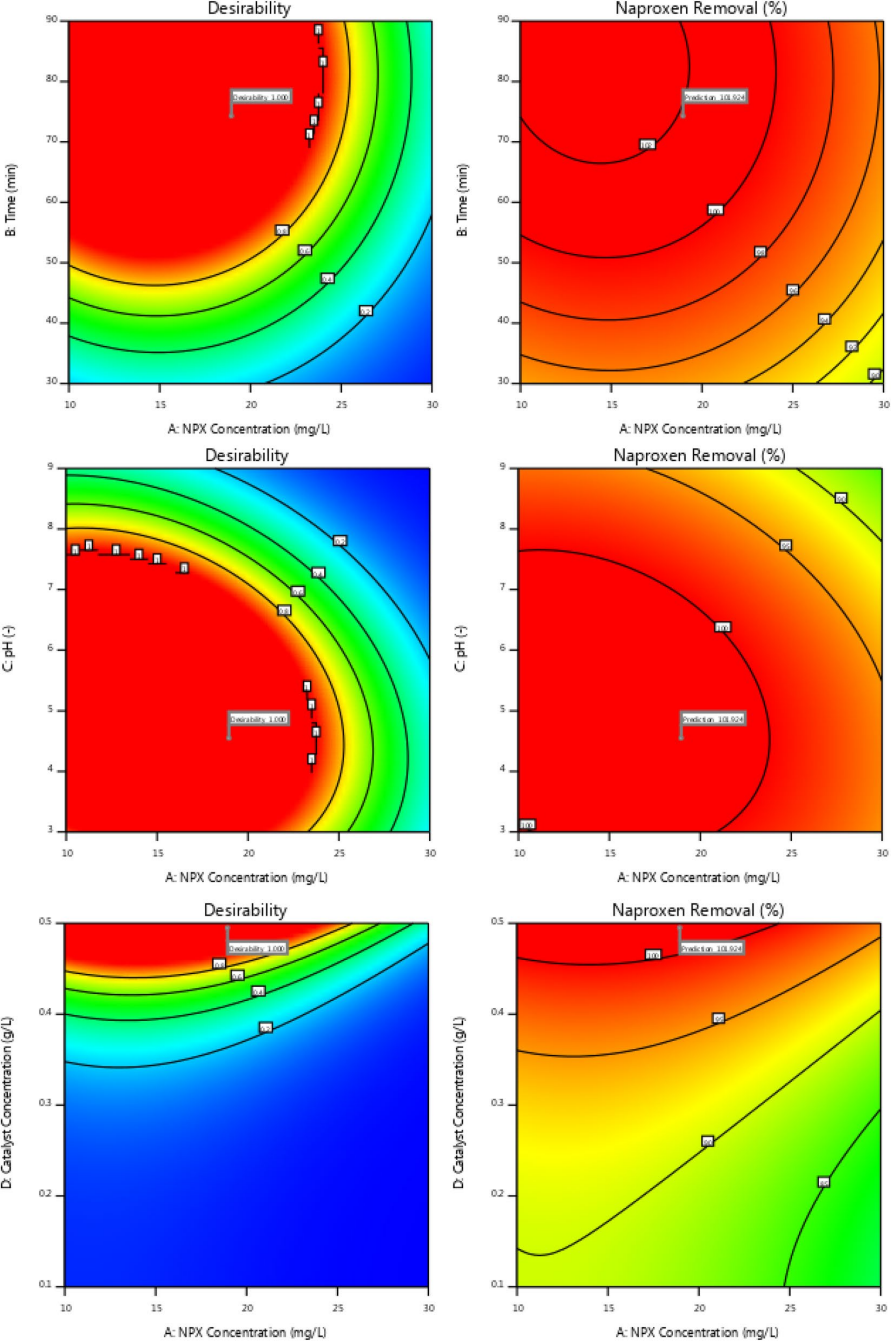
A range of independent variables of pH, photocatalyst dosage, time, and initial naproxen concentration.

An optimization was performed in order to find the optimal operating conditions for the removal of naproxen completely from pharmaceutical synthetic solutions. According to the proposed model, the following independent operating parameters are required for complete removal of naproxen (101.92%): initial naproxen concentration = 18.95 mg/L, initial pH value = 4.55, photocatalyst dosage = 0.49 g/L, time = 74.31 min. Figure 12 illustrates different operating conditions that can be used to achieve complete degradation of naproxen.

### Reusability ZrO_2_/TiO_2_/Fe_3_O_4_ photocatalyst

**Figure 13:**
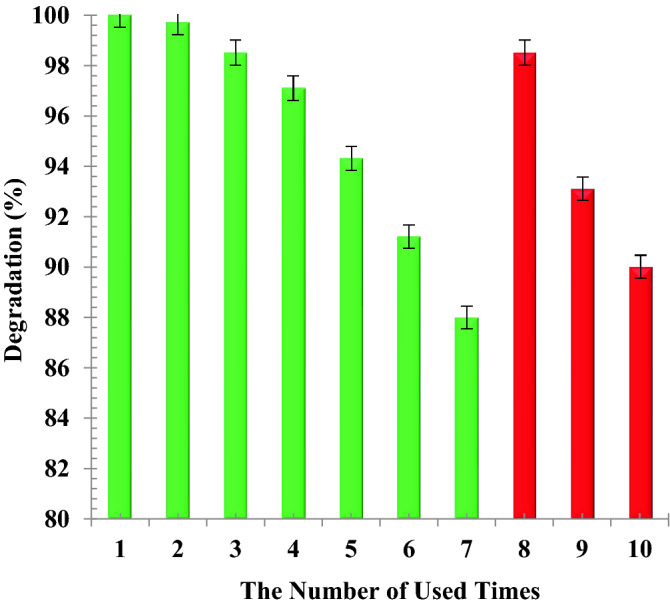
Reusability and regeneration of ZrO_2_/TiO_2_/Fe_3_O_4_ photocatalyst for naproxen degradation.

The regeneration and re-use of the used or spent photocatalyst is very critical for determining the applicability of the process applied. The used photocatalyst was separated from the synthetic solution using 1.3 Tesla magnetic. Then, it was used for the treatment of pharmaceutical synthetic solution as given in Fig. 13. As can be seen in Fig. 13, the degradation efficiency only display partial reduction after seven cycles for degradation of naproxen, indicating excellent reusability of the synthesized photocatalyst as after 7 runs, its performance for naproxen degradation was decreased only 12%. Additionally, the photocatalyst was placed in distilled water under UV light for 12 h in order to regenerate it. After doing the regeneration process, the photocatalyst was able to degrade the naproxen by about 98.4%. However, after three regenerations, the activity of the photocatalyst decreased by 8.4%. The decrease in performance of photocatalyst may be related to the blocking of active pores due to precipitation of non-sensitive chemicals to light or destroying of effective nanoparticles during the recovery process. Each experiment was replicated three times and an error bar was shown in Fig. 13.

### Analysis study of naproxen removal kinetics

**Figure 14:**
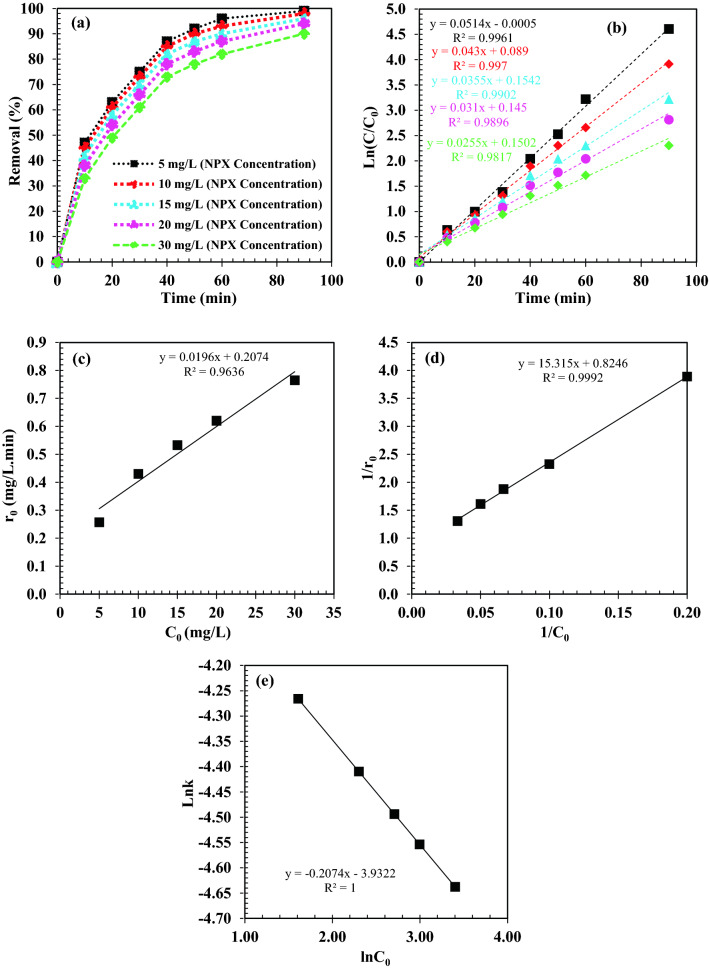
(**a**) The effect of initial concentrations on NPX removal(%), (**b**) The effect of initial concentrations of NPX on photo degradation, (**c**) The initial reaction rate (r_0_) as a function of NPX initial concentration (C_0_); (**d**) L–H model of photocatalytic NPX degradation by ZrO_2_/TiO_2_/Fe_3_O_4_ ; (**e**) The effect of different initial concentrations on the NPX photo degradation rate constant.

The removal of naproxen using heterogeneous photocatalyst is divided into two main stages: physical adsorption reaction and chemical reaction. In order to describe the photocatalytic degradation rate of naproxen by plotting ln(C_0_/C) versus time (t), at different concentrations, the Langmuir–Hinshelwood (LH) kinetic model was used^106^:

5 $$ln\frac{{C}_{i}}{C}=k.t$$

where C_i_(mg/L), the initial concentration of naproxen and C(mg/L), the concentration at time (t) after irradiation, and k is a pseudo-first-order rate constant. As shown in Fig. 14, the pseudo-first-order rate constant was determined based on a straight-line slope. According to the results of Fig. 14b, the maximum reaction rate at the initial concentration of 5 mg/L naproxen is 0.0514 min^−1^, which is about 2 times higher than the obtained reaction rate at the initial concentration of 30 mg/L naproxen. The obtained results in Fig. 14b show that reaction rates decrease with increasing concentration. The naproxen absorbed amount q_e_ (mg/g) by ZrO_2_/TiO_2_/Fe_3_O_4_ nanocomposite was calculated using the following equation (Eq. (6)):

6 $${q}_{e}=\frac{\left({C}_{i}-{C}_{e}\right).V}{m}$$

where C_i_ and C_e_ are the initial and equilibrium concentrations of naproxen (mg/L), respectively. The volume of naproxen solution (ml) is V and the mass of ZrO_2_/TiO_2_/Fe_3_O_4_ nanocomposite (mg) is m. The following equation was used to calculate the percentage of naproxen adsorption (%) (Eq. 7):

7 $$Adsorption (\%)=\frac{\left({C}_{i}-{C}_{e}\right).V}{m}$$

As shown in Fig. 14 when the initial concentration of naproxen was increased from 5 to 30 mg/L, the initial photodegradation rate (r_0_) also gradually increased from 0.26 to 0.77 mg/L. It was shown that naproxen photocatalytic degradation occurs on ZrO_2_/TiO_2_/Fe_3_O_4_ nanocomposite surfaces and that the rate of photo degradation increases as a function of increasing of adsorption. As the initial concentration of naproxen increases, the coverage of naproxen molecules on the surface of ZrO_2_/TiO_2_/Fe_3_O_4_ nanocomposite also increases accordingly. As a result, the electron transfer efficiency of naproxen molecules to the adsorbed surface and the charge produced by the light increases, which leads to an increase in the initial optical decomposition rate (r_0_). As a result, the electron transfer efficiency of naproxen molecules, which leads to an increase in the initial photodegradation rate (r_0_), increases with increasing absorbed surface and the produced charge by light. For different initial concentrations, the kinetic constant of naproxen photo degradation gradually decreased (from 0.0514 to 0.0255 min^−1^), while the R^2^ correlation coefficient also decreased from 0.9961 to 0.9817. The relationship between the reaction rate (k) and the initial concentration of the substrate during the photocatalytic process can be generally explained by the following experimental formula (Eq. 8):

8 $$k=a{[NPX]}^{n}$$

9 $$ln k=ln a+nln[NPX]$$

where n is the correlation index and [NPX] is the initial concentration (NPX) (mg.L^−1^).

Linear regression was used to analyze the relationship between the kinetic constant of NPX photo degradation and its initial concentration (5–30 mg/L). Figure 14 shows that the relationship between reaction rate k and NPX concentration is as follows:

10 $$ln k = ln 0.0196 + 0.2074.ln\left[ {NPX} \right]\quad \quad \quad \quad ({\text{R}}^{{2}} = 0.{9992})$$

The results showed that the adsorption of naproxen on the surface of ZrO_2_/TiO_2_/Fe_3_O_4_ nanoparticles is clearly time-dependent. As shown in Fig. 14a most naproxen removal occurred within the initial 50 min, and after 50 min, naproxen removals occurred almost in the flat part of the graph. The highest percentage of naproxen removal is at the initial concentration of 5 mg/L of solution. In the initial 50 min, two mechanisms were involved in the removal of naproxen: the free active sites at the adsorbent surface, and the generation of hydroxyl ions. Subsequently, a slow rate of naproxen removal was observed.

Different kinetic models were investigated to analyze the kinetic data and determine the kinetic mechanism of naproxen adsorption on the surface of ZrO_2_/TiO_2_/Fe_3_O_4_ nanocomposite which is shown in Table 6.

**Table 6:** Equations of adsorption kinetic models for naproxen adsorption on ZrO_2_/TiO_2_/Fe_3_O_4_ nanocomposite.

Model	Equation	Parameter		References
Elovich	q_t_ = (1/β)(Ln(αβ)) + (1/β)Ln(t)	R^2^	0.9202	^107–109^
		α	67.378	
		β	0.313	
Weber and Moris	q_t_ = C + K_int_(t)^1/2^	R^2^	0.9377	^107–110^
		K_int_	1.984	
		C	10.57	
Pseudo 2nd order (McKay-Ho)	(t/q_t_) = 1/(K_2_.q_e_^2^) + (1/q_e_).t	R^2^	0.9814	^111,112^
		K_2_	0.013	
		Calculated q_e_	26.4	
		Experimental q_e_	25.7	
Pseudo 1st order (Lagergeren)	Log(q_e_ − q_t_) = Log q_e_ − (K_1_/2.303).t	R^2^	0.9891	^111,113^
		K_1_	0.085	
		Calculated q_e_	17.12	

q_e_: the amount of adsorbed NPX by ZrO_2_/TiO_2_/Fe_3_O_4_ nanocomposite (mg/g) [at equilibrium].

q_t_: the amount of adsorbed NPX by ZrO_2_/TiO_2_/Fe_3_O_4_ nanocomposite (mg/g) [at determined time interval t].

K_1_: rate constant of pseudo 1st order adsorption process (min^−1^).

K_2_: the rate constant of pseudo 2nd order adsorption process (g mg^−1^ min^−1^).

α: the initial adsorption rate constant (mg/g min).

β: constant related to surface coverage and the activation energy for chemisorptions (g/mg).

K_int_: the intraparticle rate constant (mg/g min^1/2^).

C: value about the boundary thickness.

**Figure 15:**
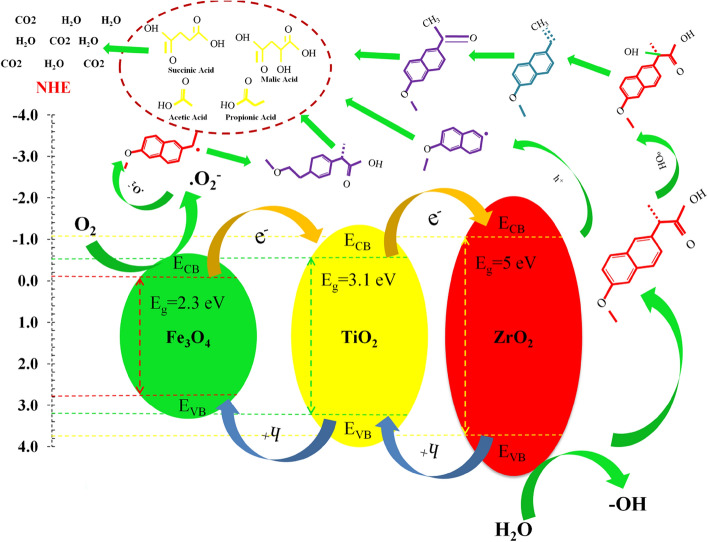
Photo degradation reaction mechanism of NPX by ZrO_2_/TiO_2_/Fe_3_O_4_.

Elovich model shows less linearity in the regression coefficient R^2^; which was found to be 0.9202, On the other hand, the correlation coefficient value for a Langmuir–Hinshelwood (LH) kinetic model was obtained 0.9992 for NPX and greater than all other adsorption kinetic models. The calculated q_e_ value was found to be 26.4 mg/g for NPX, which was very close to the obtained experimental values (shown in Table 6). According to the obtained results, it is expected that NPX and its degradation intermediates may be further degraded by reactive species, thus leading to ring openings and eventually oxidation to CO_2_ and H_2_O. As can be seen in Fig. 15, the possible degradation pathways of NPX are proposed based on the identification of intermediates and mineralization results. The photocatalytic degradation of NPX by ZrO_2_/TiO_2_/Fe_3_O_4_ mainly refers to three main pathways including decarboxylation and hydroxylation^39^. Three possible degradation pathways through oxidation processes are shown in Fig. 15. In the oxidation pathway I, NPX degradation was initiated by the electrophilic additive interaction between the naphthalene ring of NPX and ·OH^114–116^. In pathways II and III, NPX oxidation was performed by h^+^ and ·O^2−^ and carbon-based radical species were formed by decarboxylation^117^. All produced intermediates during the process were decomposed by ring-opening reactions to malic acid, succinic acid, propionic acid and acetic acid, and finally mineralized to CO_2_ and H_2_O^39,118,119^.

**Figure 16:**
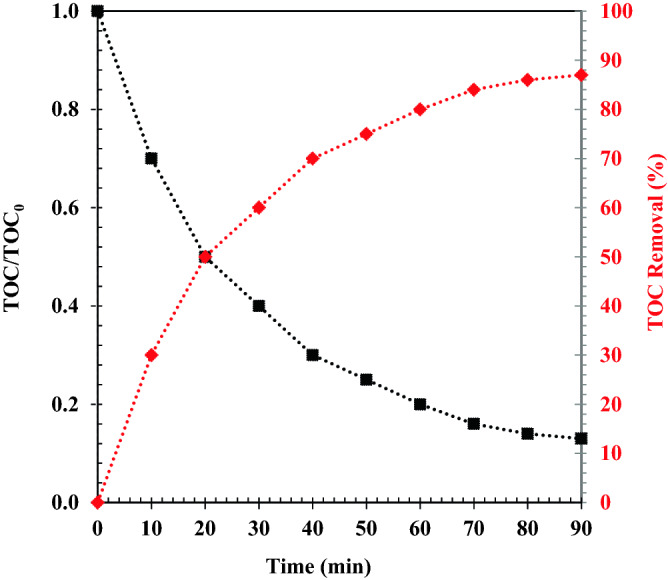
Total organic carbon (TOC) in naproxen wastewater as a function of time and the ZrO_2_/TiO_2_/Fe_3_O_4_ photocatalytic activity.

In order to confirm mineralization, we investigated total organic carbon (TOC) in the degradation of naproxen at ambient temperature and pH = 3. The amount of TOC was decreased with increasing irradiation time (Fig. 16). In this study, the mineralization of naproxen is confirmed using ZrO_2_/TiO_2_/Fe_3_O_4_ photocatalysts. TOC removal was obtained 87% after 90 min irradiation. Naproxen may be oxidized to CO_2_, H_2_O, and some small molecules, according to TOC results.

As shown in Table 7, in comparison with previous studies, the synthesized photocatalyst in this study had the highest naproxen removal efficiency. The synthesis of ternary nanocomposites confirmed the excellent synergistic effect of these three types of nanoparticles on the photocatalytic process. Based on the Table 7, the synthesized photocatalyst has high specific surface area. In addition, it’s proper capability in acidic environment increases efficiency and absorption over time for the removal of naproxen at low dosage of catalyst in comparison with other similar synthesized photo catalysts.

**Table 7:** Comparison of naproxen removal efficiency in the presence of synthesized photocatalyst under optimum process conditions.

Type of photocatalyst	Surface areas (m^2^ g^−1^)	NPX concentration (mg/L)	Irradiation time (min)	Catalyst concentration (g/L)	pH	Degradation efficiency (%)	Average pore volume (cm^3^ g^−1^)	References
ZrO_2_/TiO_2_/Fe_3_O_4_	280	10	90	0.5	3	100	0.92	Present work
TiO_2_	55	0.184	120	0.1	6.15	40	–	^120^
MoS_2_–CeO_2_–ZrO_2_	39.45	11.51	40	0.5	5.8	21	–	^121^
P_25_–TiO_2_/TEOS	–	5	600	0.003	6	94	–	^122^
HTNM	53.67	0.5	180	1.5	7	99.9	0.32	^39^
ZnO	–	40	120	0.5	7	98.7	–	^123^
Fe_3_O_4_/MWCNTs	144	10	–	0.4	7	83	0.24	^124^
N-doped TiO_2_/SiO_2_/Fe_3_O_4_	232.41	9.33	217.08	0.06	4.29	96.32	–	^125^

## Conclusion

In summary, we synthesized a ternary ZrO_2_/TiO_2_/Fe_3_O_4_ nanocomposite system using a solid-state dispersion method. Furthermore, its photocatalytic activity towards naproxen degradation was investigated. The effect of operating parameters including initial naproxen concentration, initial pH, photocatalyst dosage, and time on the degradation of naproxen was investigated. Characterization results confirmed the formation of the ternary nanocomposite. The existence of cylindrical and spherical pores in the sample was proved because its N_2_ adsorption–desorption hysteresis followed IV-H1 type hysteresis. Based on optimization results using the RSM method, the optimal conditions for the complete removal of naproxen were determined as initial naproxen concentration of 18.95 mg/L, initial pH of 4.55, photocatalyst dosage of 0.49 g/L, and time of 74.31 min. Reusability results showed 12% reduction in naproxen degradation after 7 runs. It was found that Langmuir–Hinshelwood's (LH) kinetic model was linear. The regression coefficient (R^2^) was obtained 0.9992 and it was greater than all other adsorption kinetic models.
